# The Endocytic Receptor Megalin and its Associated Proteins in Proximal Tubule Epithelial Cells 

**DOI:** 10.3390/membranes4030333

**Published:** 2014-07-11

**Authors:** Shankhajit De, Shoji Kuwahara, Akihiko Saito

**Affiliations:** 1 Division of Clinical Nephrology and Rheumatology, Niigata University Graduate School of Medical and Dental Sciences, 1-757 Asahimachi-dori, Chuo-ku, Niigata 951-8510, Japan; E-Mail: shankha1@med.niigata-u.ac.jp; 2 Department of Applied Molecular Medicine, Niigata University Graduate School of Medical and Dental Sciences, 1-757 Asahimachi-dori, Chuo-ku, Niigata 951-8510, Japan; E-Mail: kuwahara@med.niigata-u.ac.jp

**Keywords:** ARH, clathrin, ClC-5, cubilin, endocytosis, exosome, megalin, NHE3, trafficking

## Abstract

Receptor-mediated endocytosis in renal proximal tubule epithelial cells (PTECs) is important for the reabsorption and metabolization of proteins and other substances, including carrier-bound vitamins and trace elements, in glomerular filtrates. Impairment of this endocytic process results in the loss of such substances and development of proteinuria, which is an important clinical indicator of kidney diseases and is also a risk marker for cardiovascular disease. Megalin, a member of the low-density lipoprotein receptor gene family, is a multiligand receptor expressed in the apical membrane of PTECs and plays a central role in the endocytic process. Megalin interacts with various intracellular adaptor proteins for intracellular trafficking and cooperatively functions with other membrane molecules, including the cubilin-amnionless complex. Evidence suggests that megalin and the cubilin-amnionless complex are involved in the uptake of toxic substances into PTECs, which leads to the development of kidney disease. Studies of megalin and its associated molecules will be useful for future development of novel strategies for the diagnosis and treatment of kidney diseases.

## 1. Introduction

Endocytosis is a highly coordinated process that plays key roles in cell signaling and homeostasis [1]. In eukaryotes, endocytosis has long been thought of as a simple process by which cells internalize nutrients and membrane-associated molecules. Among the different types of endocytosis that have been identified to date, the most extensively studied and best characterized at the molecular level is clathrin-mediated endocytosis, which involves the internalization of cell-surface receptors and soluble molecules, including nutrients, from the extracellular fluid in clathrin-coated vesicles that bud off from the plasma membrane [2,3]. Although clathrin is the main coat component of the endocytic vesicles, a number of adaptor proteins are also involved in the initiation of vesicle budding, as clathrin is unable to interact directly with lipids and proteins in the plasma membrane [4]. These adaptor proteins are required to link clathrin with the membrane and are also able to specifically bind the endocytic cargo and ensure its uptake into clathrin-coated vesicles. After internalization, membrane receptors reach early endosome from where they are either recycled back to the plasma membrane or go to the late endosomes or multivesicular bodies (MVBs) from where they are destined to lysosomal degradation or go out of the cells in the form of exosomes. Exosomes are the nanovesicles of endocytic origin that are secreted into the extracellular space or body fluids whenever MVBs fuse with the cell membrane [5].

The receptor-mediated endocytosis and subsequent metabolization of proteins and nutrients is considered to be one of the most important functions of renal proximal tubular epithelial cells (PTECs). Receptor-mediated endocytosis requires the coordinated functioning of numerous proteins and signal transduction molecules. In particular, the membrane-associated endocytic receptor megalin and its associated proteins play a central role in the process. 

Here, we summarize the current research progress on understanding the physiological as well as pathophysiological importance of megalin and its associated molecules in the function of PTECs. 

## 2. Structure and Function of Megalin

**Figure 1:**
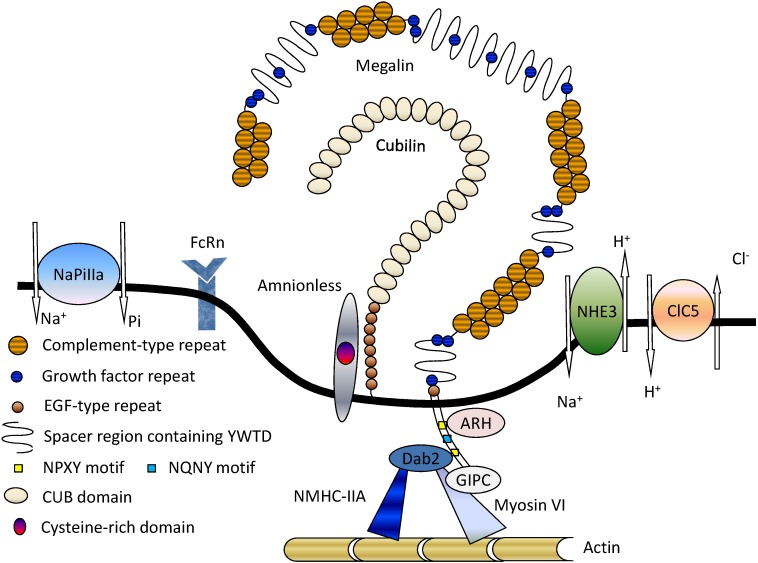
Megalin and its associated molecules in proximal tubule epithelial cells (PTECs). Among the various molecules present on the apical membrane of PTECs, megalin plays the central role in receptor-mediated endocytosis. Megalin also functions cooperatively with other membrane proteins, such as the cubilin-amnionless complex (CUBAM), NHE3, and ClC5. NaPi-IIa is responsible for the renal uptake of Pi, whereas NHE3 and ClC5 are involved in further processing of endocytosed proteins. The neonatal receptor FcRn might be involved in the uptake and transcytosis of filtered albumin. A number of intracellular adaptor proteins, including ARH, Dab2, and GIPC, are involved in megalin trafficking. Reproduced with permission from [14]. Copyright 2011 Nova Science Publishers Inc.

Megalin (gp330) was first identified as a rat Heymann nephritis antigen [6,7]. After that, the gene encoding megalin was sequenced for both rats and humans, and was mapped to chromosome 2 in humans [8,9,10]. The megalin gene encodes an extremely large glycoprotein (≈600 kDa), consisting of a large extracellular domain, small transmembrane domain, and intracellular domain, with high homology to members of the low-density lipoprotein (LDL) receptor superfamily [11]. As a common characteristic of the LDL superfamily, the extracellular domain of human megalin contains three kinds of repeats: (1) 36 cysteine-rich complement-type repeats comprising four clusters of ligand-binding domains; (2) 16 growth factor repeats that are separated by 8 YWTD-containing spacer regions and function in the pH-dependent release of ligands in endosomal compartments; and (3) a single epidermal growth factor (EGF)-like repeat [12] (Figure 1). The large extracellular domain is followed by a single transmembrane region and a cytoplasmic domain that contains two highly conserved endocytic motifs (NPXY), which interact with adaptor proteins, and an NPXY-like motif (NQNY), which is involved in the apical sorting of megalin [13]. In addition to these motifs, megalin has several other motifs with unresolved function, such as SH3 and PDZ domains, and phosphorylation sites, which are likely involved in receptor-protein interactions [8,9]. 

In the kidney, megalin is highly expressed in clathrin-coated pits and is also expressed at lower levels in PTEC microvilli [15,16]. A recent study revealed that human podocytes express megalin, which may require farther confirmation [17]. In addition to in the kidney, megalin is also expressed in different parts of the brain and central nervous system, including the brain capillaries, choroid plexus, spinal cord oligodendrocytes, astrocytes, and neurons [18,19,20,21]. Megalin is also present in intestinal brush border cells, gallbladder epithelial cells, thyroid follicular cells, ocular ciliary bodies, fallopian tubes, and uterus, and has been shown to perform various functions depending on the cell type [22,23,24,25,26].

Megalin plays a particular key role in the proximal tubular uptake of glomerular-filtered albumin and other low-molecular-weight-proteins. Consistent with this function, megalin knockout mice develop low-molecular-weight proteinuria and albuminuria [27]. Mutation of the megalin gene is associated with Donnai-Barrow syndrome, which is characterized by agenesis of the corpus callosum, congenital diaphragmatic hernia, facial dysmorphology, ocular anomalies, hearing loss, and developmental delay, and facio-oculo-acoustico-renal syndrome, which is characterized by low-molecular-weight proteinuria and albuminuria [28,29]. Megalin also interacts with sonic hedgehog and activates the associated pathway [30]. 

Several physiologically important substrates have been identified as megalin ligands including insulin [31], albumin [32], hemoglobin [33], vitamin D-binding protein (DBP) [34], retinol-binding protein (RBP) [35], β_2_-microglobulin *etc.* [31,36]. In addition, a number of toxic substances, such as glycated proteins (AGEs) [37], myeloma light chain [38,39,40], and amino glycosides [41], also interact with megalin and undergo endocytosis, leading to PTEC injury. A complete list of megalin and cubilin ligands is given in the review by Christensen *et al.* [42]. 

## 3. Megalin-Associated Molecules

### 3.1. Cubilin

Cubilin, which was first identified as the receptor for intrinsic factor-vitamin B_12_, is co-expressed with megalin on apical epithelial cells [43,44]. Mutations in the cubilin-encoding gene cause hereditary megaloblastic anaemia 1 and Imerslund-Gräsbeck syndrome, which are associated with selective vitamin B_12_ malabsorption and proteinuria [45]. Recently, human and rat glomerular podocytes were also shown to express cubilin [46]. This 460 kDa peripheral membrane glycoprotein lacks a transmembrane as well as intracellular domain, but anchored via its NH_2_ terminus to apical membranes in PTECs [47]. Cubilin consists of 27 CUB (complement components C1r/C1s, Uegf, and bone morphogenic protein 1) domain cluster preceded by 8 EGF repeats and a short, 110-amino-acid *N*-terminal sequence [48]. Cubilin also functions as an endocytic receptor by binding to various ligands, including albumin [49], transferrin [50], DBP [51] *etc.*, present in glomerular filtrates. As cubilin lacks an intracellular domain, it is thought to require interaction with megalin for proper functioning [52]. However, cubilin forms a functional complex with amnionless (AMN) named CUBAM, which is translocated to the plasma membrane and displays megalin-independent activity [53,54]. AMN is a 38–50 kDa membrane protein that contains a 70-amino-acid extracellular domain with a cysteine-rich domain and a cytoplasmic tail with 2 NPXY motifs [55]. AMN interacts with the EGF domains of cubilin to facilitate export of the CUBAM complex from the endoplasmic reticulum and for membrane attachment [56]. Recently, CUBAM-mediated protein reabsorption has been observed in *Drosophila* nephrocytes, which play a similar role in filtration as mammalian glomerular podocytes [57].

### 3.2. Na^+^/H^+^ Exchanger Isoform 3 (NHE3)

Among the different isoforms of Na^+^/H^+^ exchanger (NHE), NHE3 is predominantly found in PTECs, where it mediates the isotonic reabsorption of NaCl [58] and NaHCO_3_ [59], and the secretion of ammonium [60,61]. NHE3^−^/^−^ mice exhibit decreased cortical bone mineral density and trabecular bone mass, demonstrating that epithelial NHE3 is necessary for renal and intestinal calcium reabsorption [62]. NHE3 is also involved in the reabsorption of citrate, amino acids, and oligopeptides from the urinary filtrate. Enhanced NHE3 activity may be responsible for elevated Na^+^ retention and increased susceptibility to hypertension, as suggested by the enhanced Na^+^/H^+^ exchange activity of NHE3 in PTECs of spontaneous hypertensive rats [63]. It is possible that a post-transcriptional event(s) leads to the increased expression and activity of NHE3, because mRNA levels were not elevated [63]. 

NHE3 interacts with megalin in the intermicrovillar clefts of PTECs [64,65]. The importance of NHE3 in Na^+^ and acid-base homeostasis is exemplified by the hypovolemic hypotension and metabolic acidosis seen in NHE3^−^/^−^ mice despite compensation by the distal nephron [66]. The abundance of NHE3 in PTECs, and its specific activity and subcellular localization are altered in rats with puromycin aminonucleoside (PAN)-induced nephrotic syndrome, suggesting a novel mechanism for the control of Na^+^/H^+^ exchange *in vivo* [67]. In PTECs, insulin regulates the volume and acid-base balance through stimulation of NHE3 via yet undefined mechanisms, although the chronic effect of insulin is generally mediated by the classical PI3K-SGK1 pathway [68]. Endothelin increases acidification of acid-challenged animals and PTECs through enhanced activity of NHE3 [69]. After endocytosis with megalin, NHE3 is postulated to utilize the outward transvesicular Na^+^ gradient of endocytic vesicles and early endosomes to drive the inward H^+^ movement required for endosomal acidification, which is an important step in megalin trafficking [61].

### 3.3. 2Cl^−^/H^+^-Exchanger (ClC-5)

ClC-5 is 746-amino-acid protein that was originally identified as a member of the voltage-gated chloride channel family [70], but later shown to function as an H^+^/2Cl^−^ exchanger [71]. In the kidney, ClC-5 is highly expressed in PTECs, and α- and β-intercalated cells of collecting ducts [72]. Recently, it has been found that ClC-5 is expressed in human podocytes [73]. Similar to its closely related homologs ClC-3 and ClC-4, ClC-5 is primarily located on endosomal membranes; however, it is also expressed a low levels on plasma membranes [74,75]. In apical endosomes, ClC-5 functions in endosomal acidification in conjunction with electrogenic V-type H^+^-ATPases, where they are co-localized [72,74]. Evidence suggests that ClC-5 provides an electric shunt for vesicular H^+^-ATPase (12–15), which is required for efficient endosomal acidification [76,77]. ClC-5 has a unique role in renal endocytosis among ClC exchangers and does not require PY-motif-dependent ubiquitylation [78]. ClC-5 and kinesin family member 3B (KIF3B), a heterotrimeric motor protein that facilitates fast anterograde translocation of membranous organelles, interact directly in polarized renal PTECs, which reabsorb proteins and solutes via the megalin and cubilin receptor-mediated endocytic pathway. The interaction between ClC-5 and KIF3B *in vivo* leads to altered ClC-5 cell-surface expression, microtubular transport, and endocytosis of ClC-5-containing vesicles from the cell surface of polarized epithelial cells [79]. ClC-5 interacts with megalin in conjunction with a regulatory factor called Na^+^/H^+^ exchanger regulatory factor 2 (NHERF2), which acts as a scaffold for the two proteins [80]. The particular role of ClC-5 in megalin and cubilin-mediated endocytosis was confirmed when in ClC-5 knockout mice showed a reduced endocytosis of megalin/cubilin ligands, confirming that ClC-5 is required for the trafficking of megalin and cubilin in PTECs [81]. In contrast to the kidney, ClC-5 does not affect the expression or function of megalin in the thyroid gland, suggesting that megalin might be regulated differently in these two organs [82].

Mutations of the human gene encoding ClC-5 (*CLCN5* gene) cause the X-linked disorder of the proximal tubules known as Dent’s disease, which is characterized by low-molecular-weight proteinuria, hypercalciuria, nephrolithiasis, aminoaciduria, phosphaturia, and renal failure [83,84]. The pathology may result from defective fluid-phase and receptor-mediated endocytosis, and endosomal acidification, and/or reduced expression of megalin and cubilin in PTECs due to *CLCN5* gene disruption [85,86]. Genomic analysis of a patient with Dent’s disease and his family revealed a novel glycine-to-arginine transition mutation at the first nucleotide of codon 333 of *CLCN5*, which was associated with the markedly reduced and irregular expression of megalin and cubilin as well as adaptor protein Dab2 compared to a control subject [87]. Despite the identification of this mutation, Dent’s disease exhibits genetic heterogeneity, with approximately 50%–60% of patients having *CLCN5* mutations (Dent’s disease 1), approximately 15% harboring *OCRL1* mutations (Dent’s disease 2) and the remaining 25%–35% of patients having neither *CLCN5* nor *OCRL1* mutations, but possibly having defects in other genes [88]. Recently, an atypical Dent’s disease phenotype was found to be due to co-inheritance of mutations in both *CLCN5* and *OCRL* [89].

### 3.4. Type IIa Sodium Phosphate Co-Transporter (NaPi-IIa)

Renal reabsorption of phosphate (Pi) is essential for maintaining plasma Pi homeostasis. Among the different types of Na^+^-dependent-Pi-co-transporters identified to date, type II is the functionally dominant type in renal PTECs [90]. The importance of this co-transporter was revealed in mice when targeted inactivation of the *Npt2* gene caused 70% less renal reabsorption of Pi, leading to renal Pi wasting, hypercalciuria, and skeletal abnormalities. [91] The basic mechanism of Pi transport across the proximal tubular and small intestinal brush borders is nearly identical, but involves NaPi-IIa and NaPi-IIb co-transporters in the kidney and gut, respectively [92]. Renal reabsorption of Pi is mediated by NaPi-IIa on PTECs and altered expression of this co-transporter in the brush border region leads to differences in renal Pi transport [93]. Parathyroid hormone (PTH) induces the inactivation of NaPi-IIa by promoting its endocytic retrieval and degradation, and it has been shown that megalin facilitates this process. Steady-state expression and PTH-driven inactivation of NaPi-IIa in PTECs is megalin dependent [94]. NaPi-IIa is co-expressed with NHE3 at apical microvilli; however, NHE3 is present within lipid rafts, whereas NaPi-IIa is predominantly located in nonrafts. In addition, kidney-specific inactivation of the megalin gene impairs trafficking of renal NaPi-IIa [94,95]. Analysis of the rat renal cortex revealed that NaPi-IIa heterogeneously co-localizes with ezrin and megalin on the apical membrane of PTECs [96]. In response to hormones and a high dietary Pi content, NaPi-IIa is endocytosed and then either degraded in lysosomes or targeted to the trans-Golgi network (TGN), where it interacts with PIST (PDZ-domain protein interacting specifically with TC10), a TGN-resident PDZ-domain-containing protein [97]. Recently, fibroblast growth factor-23 (FGF23) was found to contribute to several hypophosphatemic disorders by reducing the expression of NaPi-IIa and NaPi-IIc in PTECs, as well as by lowering serum 1,25-dihydroxyvitamin D(3) levels [98].

### 3.5. FcRn

The neonatal Fc receptor for IgG (FcRn) is responsible for the transfer of passive humoral immunity from a mother to her fetus, and also performs diverse functions in various adult tissues [99]. FcRn is comprised of β_2_-microglobulin and a membrane-anchored α-chain related to major histocompatibility complex class I (MHC-I) [100,101]. The pH-dependent interaction between FcRn and albumin, which is stronger at lower pH, is similar to that observed between FcRn and IgG and is critical for recycling albumin back into the circulation and avoiding cellular degradation, thereby prolonging its serum half-life [102]. Despite the overall similarity in the pH-dependent binding mechanism with that of IgG, albumin binds FcRn with a 1:1 stoichiometry and the interaction is hydrophobic in nature, suggesting that the FcRn-albumin interaction is distinct from FcRn-IgG binding [103]. In contrast, Studies of opossum kidney-derived cultured PTECs have demonstrated the FcRn-independent megalin- and/or cubilin-mediated uptake of FITC-conjugated IgG [104]. In the kidney, FcRn is expressed in podocytes and the brush border of PTECs [105]. In podocytes, FcRn is involved in the transcytosis of IgG around the slit diaphragm and into the urinary space, where it is then reabsorbed by FcRn in proximal tubules [106]. Recently, it was reported that FcRn^−^/^−^ mice have a lower t_1⁄2_ for albumin compared to wild-type mice, suggesting that renal FcRn reclaims albumin, but facilitates the loss of IgG from plasma protein pools [107]. Very recently, Tenten *et al.* proposed that the transcytosis of filtered albumin, as well as IgG, is FcRn dependent [108]; however, the physiological and pathophysiological role of the transtubular transport of filtered intact albumin is not yet known [109]. Further investigations are needed for a clearer understanding of the tubular processing of albumin by FcRn receptors. 

### 3.6. Intracellular Adaptor Proteins

JIP1, JIP2, SEMCAP-1 (GIPC), ANKRA, PDS-95, MegBP, *etc.* are intracellular adaptor proteins that bind the cytoplasmic tail of megalin. Although these adaptor proteins are thought to facilitate endocytosis, their individual roles in megalin trafficking remain unclear [110,111,112,113,114]. Of these adaptor proteins, Dab2 and Autosomal Recessive Hypercholesterolemia (ARH) have been the most extensively studied and their roles in megalin trafficking are described below.

## 4. Megalin Trafficking and Expression in PTECs

### 4.1. Role of Adaptor Proteins in Megalin Trafficking

The complete mechanism and roles of megalin-associated adaptor proteins in megalin trafficking are largely unknown. In general, endocytosis of all LDL receptor family members require crucial NPXY signal in the cytoplasmic tail [115] and megalin also interacts with a number of proteins via its two conserved NPXY motifs present in its cytoplasmic domain. First NPXY motif of megalin binds to the phosphotyrosine-binding (PTB) domain of the ARH [116], and the second NPXY motif was shown to interact with the PTB domain of Dab2 [117] and both of them are considered to be clathrin-associated sorting proteins [118].

The adapter protein Dab2 and megalin mutually regulate each other’s localization in PTECs. The expression of Dab2 in PTECs appears to be dependent on megalin or factors associated with megalin, while knockingout of the Dab2 gene decreases the level and alters the subcellular distribution of megalin in PTECs [119]. In addition to PTECs, Dab2 is also required for the endocytosis of megalin by visceral endoderm cells [120] and plays a major role in LDL receptor internalization in HeLa cells and fibroblasts [121]. Notably, Dab2 mediates internalization of LDL receptor family, but not transferrin receptor, and functions independently of ARH and AP-2 (classic clathrin adaptor protein) [121]. Dab2 and adapter protein GIPC facilitates binding of the megalin cytoplasmic tail to the reverse-direction molecular motor myosin VI, an interaction that is thought to be crucial for endocytosis in PTECs [122]. However, myosin VI knockingout mice show no significant renal manifestations or proteinuria [123]. In our studies, we identified another motor protein, nonmuscle myosin heavy chain IIA (NMHC-IIA) that binds to Dab2 and is involved in the endocytic process [124]. Genetic alterations to the NMHC-IIA gene are associated with the inherited human disease *MYH9* disorder, which is characterized by giant platelets, thrombocytopenia, and granulocyte inclusions [125,126,127], thus indicating the importance of NMHC-IIA in maintaining normal kidney function. The involvement of NMHC-IIA in this disorder was verified in two genome-wide scan analyses of *MYH9* patients [128,129]. More recently, it was demonstrated that protein kinase B (PKB)/Akt, which forms part of the endocytic machinery, mediates albumin uptake through interaction with Dab2 [130]. 

**Figure 2:**
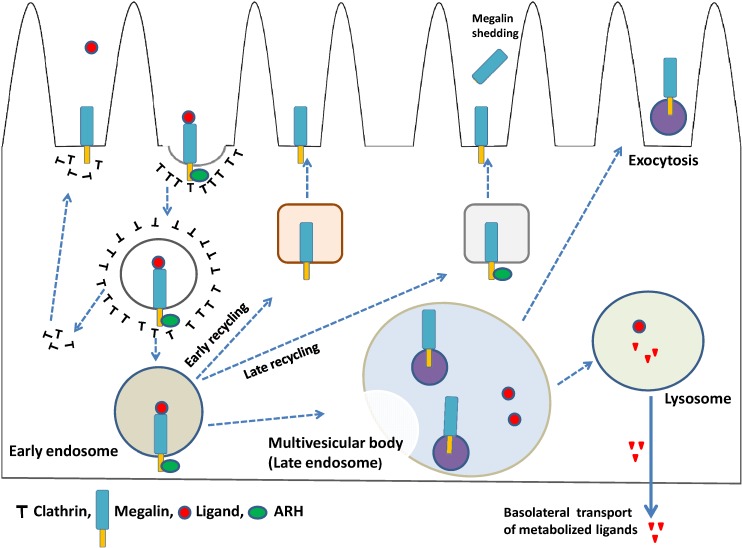
Megalin-mediated endocytosis and megalin trafficking. The plasma membranes start to invaginate and at last bud off during endocytic internalization and reach early endosomes, where the decision for further ligand processing is made depending on the cargo content. In the absence of ARH, megalin is recycled back to the plasma membrane via the early recycling pathway, whereas in the presence of ARH, megalin is recycled back to the membrane via the late recycling pathway. Depending on the specific protein interactions and domain-based sorting processes, early endosomes may also be directed to late endosomes, which then matures into a late endosomal compartment and forms multivesicular bodies. When multivesicular bodies come into contact with the plasma membrane, their contents are secreted in the form of exosomes. Megalin ligands may be subjected to lysosomal degradation and the metabolized ligands then undergo basolateral transcytosis. Megalin also undergoes regulated intramembrane proteolysis (RIP) and is shedded from plasma membrane.

The potential role of ARH in LDL receptor function was first proposed when autosomal recessive hypercholesterolemia was found to be caused by mutations in the ARH gene [131]. ARH is a protein that binds to clathrin, AP-2, and the NPXY motif of megalin [132,133]. ARH facilitates the endocytosis and trafficking of megalin along the endocytic pathway [116], and also has been shown to associate with motor and centrosomal proteins, and to play a role in centrosomal assembly and cytokinesis [134]. In a recent study, it is reported that ARH is required for the trafficking of megalin from early endosomes to the endosomal recycling compartment by coupling with dynein; however, in the absence of ARH, megalin returns directly to the cell surface from early endosomes via the connecdenn2/Rab35 fast recycling pathway (Figure 2). ARH-mediated trafficking of megalin is necessary for γ-secretase-mediated cleavage of megalin and release of a tail fragment that mediates transcriptional repression of megalin mRNA [135]. In addition, ARH cooperates with epithelial cell-specific clathrin adaptor protein AP-1B in the basolateral exocytosis of LDL receptor family from recycling endosomes in polarized epithelial cells [136]. 

The adaptor proteins ARH and Dab2 are not only crucial for megalin trafficking, but are also essential for endocytosis of the endocytic receptor CUBAM. Like the LDL receptor superfamily, cubilin contains a ligand-binding domain while AMN provides membrane anchorage and potential endocytic capacity via two NPXY signals via ARH and Dab2 [137]. Future studies are required to elucidate the complete set of interactions and adapter proteins underlying megalin trafficking. 

### 4.2. Megalin-Expressing Exosomes

Although exosomes have been described since 1981, they received relatively little attention from the scientific community until the past decade. Shortly after the discovery of exosomes, an electron microscopic study of sheep erythrocytes reported that exocytosis results in vesicles of approximately 50-nm in size [138]. Exosomes are membrane vesicles of endocytic origin and are released by different types of cells upon the fusion of multivesicular bodies with the plasma membrane [139] and should not be confused with microvesicles, which bud directly from the plasma membrane into the extracellular space upon certain stimulation [140]. Exosomes range in size from 30–120 nm, [138] and are released by different cell types under physiological, as well as pathophysiological, conditions [141]. Because exosomes can contain various cellular components, including proteins, mRNAs, and miRNAs, and are produced by one cell but taken up by other cells, they are thought to represent a new mode of intercellular communication that can modulate diverse physiological and pathophysiological phenomenon, such as angiogenesis, immunoreactions, and cell proliferation [142,143]. 

Urinary exosomes, which are released by epithelial cells facing the urinary space, are considered to be a promising source of molecular markers for renal dysfunction and structural pathology [144]. Proteomics studies have revealed that urinary exosomes contain the endocytic receptors megalin and cubilin, in addition to several other important markers and transporter proteins, such as NHE3, sodium-glucose co-transporter 1, aquaporin-1 from proximal tubules, sodium-potassium-chloride co-transporter 2 from the thick ascending limb, and thiazide-sensitive sodium-chloride co-transporter from the distal convoluted tubule [145,146]. Thus, the study of exosomal proteins, as well as associated mRNAs and miRNAs, is expected to provide greater understanding of the molecular mechanisms of diseases and identify related biomarkers [147]. A schematic diagram of megalin-expressing exosomes from PTECs is shown in Figure 2. 

### 4.3. Megalin Expression and Regulated Intramembrane Proteolysis (RIP)

TGF-ß was found to downregulate the cellular expression of megalin [148], whereas insulin and high-concentration glucose (17.5 mM) upregulate megalin expression in opossum kidney-derived cultured PTECs [148]. Megalin is also downregulated by angiotensin II and competitive cross-talk occurs between anigotensin II type 1 receptor and insulin-induce megalin expression, which serve as a counterbalancing mechanism for the regulation of megalin expression and function in PTECs [149]. We also demonstrated that low levels of lipopolysaccharide initiate the TNF-α-ERK1/2 signaling pathway, which is involved in the downregulation of megalin expression in cultured PTECs [150]. In contrast, peroxisome proliferator-activated receptors (PPARs) α and γ, which are transcription factors belonging to the nuclear receptor superfamily, and PPAR α and γ agonists positively control megalin expression [151]. Bardoxolone methyl, a potent activator of the nuclear factor erythroid 2-related factor 2 (Nrf2), also decreases megalin expression, but not cubilin expression, in monkey PTECs [152]. However, further studies are required to understand the molecular mechanisms related to megalin expression in the presence of bardoxolone methyl.

Similar to other large transmembrane proteins, such as notch receptors, megalin also undergoes RIP, which produces the megalin intracellular domain (MICD). In this process, megalin is subjected to PKC-regulated, metalloprotease-mediated ectodomain shedding, producing a membrane-bound, 35–40 kDa COOH-terminal fragment, which is further processed by γ-secretase to produce a soluble MICD [153]. In cultured opossum kidney-derived PTECs, megalin COOH-terminal domains regulate expression of the megalin gene, as well as NHE3 [154]. Notably, however, the soluble intracellular domain of megalin appears to have no role in the regulation of gene expression in PTECs *in vivo* [155].

## 5. Megalin-Mediated PTEC Injury

Overloaded endocytosis in PTECs might cause tubulointerstitial injury in various pathogenic conditions. Megalin has been identified as the key molecule in the initiation of the pathogenic process of endocytosis-mediated PTEC injury [156]. In particular, albumin overload in PTECs induces oxidative stress, the upregulation of stress-related genes [157], and activation of NF-ƙβ, which is responsible for enhancing the synthesis of the inflammatory chemokine RANTES and production reactive oxygen species that serve as secondary messengers of NF-ƙβ activation [158]. Metabolically overloaded PTECs are also activated to express a number of pro-inflammatory cytokines, such as MCP1 and TNFα and leading to apoptosis [158] or epithelial-mesenchymal transition (EMT) [159,160]. In diabetic patients, megalin mediates the endocytosis of glomerular-filtered AGEs by PTECs [37,161], leading to cellular toxicity [162]. The intake of AGEs might also overload megalin and lower cobalamin uptake, which might cause the intracellular cobalamin deficiency that is often observed renal dysfunction, diabetes, and aging. Shedding of the megalin and transcobalamin receptors under glycated conditions has also been observed [163]. Megalin-mediated endocytosis of myeloma light chain into PTECs initiates a number of inflammatory processes, resulting in cell toxicity and EMT which are the underlying cause of chronic tubulointerstitial diseases and acute renal injury in myeloma [164,165,166]. Receptor-associated protein (RAP), which is the ligand as well as chaperone protein of megalin, can block internalization and toxicity of myeloma light chain in cultured PTECs [40]. Taken together, this evidence clearly demonstrates that megalin plays a critical role in the normal physiology, as well as pathophysiology, of PTECs. 

We have established an ELISA system to quantitate full length and ectodomain forms of megalin in human urine, which will be useful for the clinical evaluation of PTEC injury [167]. Further studies are needed to elucidate the complete molecular mechanism and develop strategies for preventing PTEC damage.

## 6. Conclusions

Megalin is an endocytic receptor involved in the reabsorption of nutrients, carrier-bound vitamins, and trace elements from glomerular filtrates via interaction with various molecules in PTECs. This receptor also mediates the renal uptake of pathological substances or the overloaded endocytosis that can cause cellular damage. Megalin-mediated signal transduction might also be involved in this process. Further studies are required to elucidate the complete molecular mechanism underlying tubulointerstitial injury and develop novel strategies for preventing PTEC damage and diagnosing and treating different kidney diseases.
